# Promoting wellbeing and empowerment via Youth First: Exploring psychosocial outcomes of a school-based resilience intervention in Bihar, India

**DOI:** 10.3389/fpsyt.2022.1021892

**Published:** 2022-11-17

**Authors:** Katherine S. Leventhal, Peter L. Cooper, Lisa M. DeMaria, Priyadarshi Priyam, Hari Shanker, Gracy Andrew, Steve Leventhal

**Affiliations:** ^1^CorStone, Baltimore, MD, United States; ^2^Department of Clinical Psychology, School of Health in Social Science, University of Edinburgh, Edinburgh, United Kingdom; ^3^International Division, Mathematica, Princeton, NJ, United States; ^4^CorStone India Foundation, New Delhi, India

**Keywords:** India, wellbeing, empowerment, mental health, adolescents, schools, health promotion

## Abstract

Youth worldwide are struggling with increased mental health concerns. As youth in low- and middle-income countries make up more than 20% of the world’s population, finding ways to improve their psychosocial wellbeing is crucial. CorStone’s Youth First program is a school-based psychosocial resilience program that seeks to improve the mental, physical, social, and educational wellbeing of early adolescents. The program is delivered via trained government schoolteachers who facilitate students’ learning and development in small groups using a discussion and activity-based curriculum. In August 2021, a study among 322 adolescents was conducted to investigate and compare program participants’ and non- participants’ understanding and use of inter- and intra-personal psychosocial skills. Focus group discussions were held with students in eight intervention schools and four comparable schools not receiving the intervention (control). Through the focus group discussions, students provided their opinions, thoughts, and ideas about vignettes describing challenges that youth in their communities frequently face, including early marriage and financial pressures. Analysis integrated qualitative and quantitative approaches, consisting of an iterative thematic analysis process followed by quantizing data and conducting *t*-tests. Youth who had received Youth First had greater awareness of problems, perspective-taking, problem-solving strategies, helping approaches, awareness of their own strengths, and visions for the future, when compared with the control group. Findings provide insights into potential outcomes for measurement in future evaluations of mental health promotion and prevention programs among youth in low- and middle-income countries.

## Introduction

Threats to psychosocial wellbeing are significant and growing for youth worldwide. Mental and substance use disorders are the leading causes of disability for children and youth around the world (1), and rates of disorder have increased in recent years. For instance, rates of anxiety and depression among youth have doubled during the COVID-19 pandemic (2), and growing concerns related to climate change have created rising eco-anxiety, particularly among youth (3). Those facing poverty and social inequalities, including youth in low- and middle-income countries (LMICs), are particularly vulnerable, as resource scarcity and inequality represent significant social determinants of mental health (e.g., 4, 5). Finding ways to support youth to navigate these issues is urgent, such that they retain and/or improve their psychosocial wellbeing in the face of challenges. In addition, it is important to find ways to change – and/or to support youth to participate in changing – the circumstances and unequal social structures that are deleterious to psychosocial wellbeing in the first place (6).

One promising way to support youth psychosocial wellbeing, particularly in the face of challenges, is via preventive and promotive mental health programs for youth, including resilience-based programs (7, 8). Tailoring these programs to school-based delivery may increase the potential for school systems to institutionalize resilience programs in the future, thus increasing reach and scalability (9). Programs that integrate attention to social inequalities are also particularly important as they can support youth in challenging the *status quo* (10, 11).

Although there is growing evidence supporting the efficacy of preventive and promotive mental health interventions among youth, and particularly those implemented in schools, most interventions and evaluations have been conducted in higher income country (HIC) settings, with fewer conducted in LMIC settings (11–13). As youth (ages 10−24) in LMICs make up more than 20% of the world’s population (14), establishing which interventions work to improve their psychosocial wellbeing represents a key area of study.

In addition, another barrier that impedes our understanding of which preventive and promotive mental health programs work in LMIC school settings is that doing so requires quantifying program effects. However, when researchers seek to quantify effects of these programs, they must rely on knowledge originating via HIC studies and construct definitions, which can be problematic. Mental health promotion and prevention programs have been shown to have a diverse multitude of beneficial effects across multiple life domains (12, 15), but outcomes are always strategically selected for evaluations. These selections are generally made *a priori*, based on theory, questions, and effects that have been observed in similar programs and studies, many of which have been conducted in HICs. Outcome selection decisions may also be influenced by which measures are readily available, which also have often been developed in HICs. It is possible, therefore, that program evaluations that seek to quantify the effects of preventive and promotive mental health programs in LMIC schools may measure outcomes that do not map well to outcomes that are actually achieved. If so, this discrepancy between outcomes measured vs. outcomes achieved may result in incomplete understandings of the effects of such interventions in LMICs.

In this study, we used qualitative and quantitative methods to shed light on this issue: we intended to qualitatively describe outcomes of a school-based mental health promotion and prevention program, Youth First, via the voices and insights of LMIC adolescent participants themselves, and quantitatively compare the insights of participants vs. non-participants. The study, conducted in Bihar, India, observed Youth First participants’ inter- and intra-personal psychosocial skills and attitudes, defined broadly, contrasting these skills and attitudes with those of a control group that did not attend Youth First. This approach allowed us to highlight outcome categories that emerged inductively within the larger category of inter- and intra-personal psychosocial skills and attitudes, thus providing insights that can be useful to future researchers who seek to quantify the psychosocial effects of such programs. This paper reviews findings from the study and applies its implications to a set of broader recommendations for researchers conducting outcome and measure selection in studies that aim to quantify school-based mental health promotion and prevention program effects among LMIC youth.

### About Youth First

CorStone’s Youth First program is a psychosocial resilience, adolescent health, and gender rights intervention, which aims to promote the psychosocial, physical, and educational wellbeing of its participants. It is developed specifically for early adolescents in LMICs and has been implemented in India, Kenya, and Rwanda. The program is designed to be conducted in schools by teachers within the school day via weekly hour-long facilitated small group sessions. In Bihar, India, where Youth First has been implemented since 2013, Youth First is a 2-year program, conducted in government schools and facilitated by government schoolteachers among their 7th and 8th Standard students (equivalent to United States 7th and 8th grades). The time and human resources required to implement, oversee, and sustain Youth First are aligned with those that would reasonably be available in the Bihar government school system, thus supporting the potential for Youth First’s future institutionalization within this and similar systems.

**FIGURE 1:**
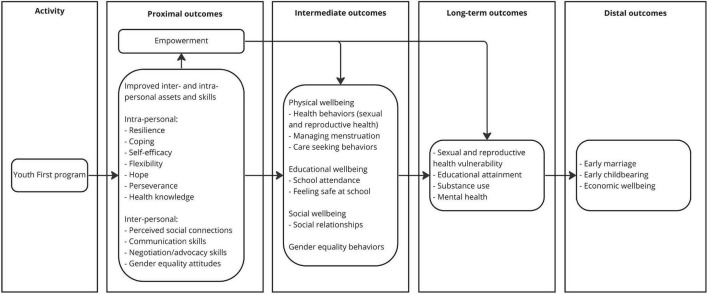
Youth First theory of change.

Youth First is assumed to work by improving inter- and intra-personal assets and skills as proximal outcomes, such as coping skills, hope, flexibility, perseverance, communication skills, and gender equality attitudes. It is additionally assumed that building higher levels of these assets, skills, and attitudes, i.e., assets, skills and attitudes that are applied more broadly and consistently across life domains and integrate greater complexity and nuance, will be related to greater improvements in outcomes that are predicted by these proximal outcomes. The proximal outcomes then bolster intermediate outcomes of physical, educational, and social wellbeing and behavior change. In addition to an expected direct relationship between proximal and intermediate outcomes, empowerment is expected to mediate the relationship. Over the long term, further effects are expected, including distal changes in economic situation and age at marriage and childbearing. See Figure 1 for the Youth First theory of change, describing expected proximal, intermediate, long-term, and distal outcomes.

In a previous randomized controlled trial (RCT) among middle school girls in Bihar, India, Youth First improved emotional resilience, self-efficacy, social-emotional assets, psychological wellbeing, and social wellbeing (16), as well as physical health knowledge, gender equality attitudes, clean water behaviors, hand washing, menstrual hygiene, health communication, ability to get to a doctor when needed, substance use, safety, and physical vitality and functioning (17) vs. controls (at the time, the program was called Girls First). As with many studies regarding psychosocial resilience programs, however, this previous RCT identified specific program outcomes *a priori* (i.e., before the trial began), by defining target outcomes and associated measures at the start. The qualitative component of that study, conducted after the intervention and without a control group, suggested other program effects that had not been measured via the quantitative scales in the study, including effects on empowerment, early marriage, problem solving skills, and coping skills (18). The current study builds upon these findings by introducing a control group and relying on vignette-based qualitative data collection methods, allowing for an open and robust examination of potential program effects.

## Materials and methods

The study aimed to investigate *how* and *whether* students who participated in Youth First displayed Youth First’s proximal outcomes – inter- and intra-personal psychosocial skills and assets – vs. students who had not participated.

Undertaken as part of a mixed-methods cluster-randomized controlled trial (cRCT) of Youth First, 70 schools were randomly assigned to either an intervention arm that received the Youth First curriculum or a control arm that received standard education. The control arm also received modest donations of art supplies, school supplies, and small classroom needs (e.g., carpets and dustbins).

The current study focuses on Focus Group Discussion (FGD) data gathered from students who attended an intervention or control school during the intervention period of 2018−2020. FGD data included in this study were collected from August to September 2021, 1.5 years after the completion of the Youth First program.

### Methodological underpinnings

This study, although its data source would traditionally be considered qualitative (FGDs), draws upon a combination of variance theory (generally associated with quantitative approaches) and process theory (generally associated with qualitative approaches) (19). As many have written, the boundaries between qualitative and quantitative approaches are often blurry (20); this study represents one such case. In alignment with process theory, we seek to understand *how* attending the intervention (Youth First) is associated with differences in observed skills and attitudes; in alignment with variance theory, we seek to understand *whether* attending the intervention is associated with differences in observed skills and attitudes (21). To do so, we combine qualitative and quantitative methods of analysis.

To understand *how* Youth First is associated with differences in observed skills and attitudes, we employed FGDs and iterative thematic analysis, both deductive and inductive. Qualitative methods are well-suited to questions regarding how and why phenomena are observed, thus making qualitative approaches appropriate for this component of the methods (19). As we define impact following Ross, as “open, broad, and reflective of a broad swath of changes that might be attributed to some program or intervention, over a significant length of time, and in multiple areas” (22) (p. 4), using qualitative methods allowed observed patterns and outcomes to be explored and explained richly, without adhering to narrow, pre-defined success indicators (22). This study therefore follows in the footsteps of scholars who have increasingly called for qualitative data and analysis to take a more significant role in discussions of “impact” (e.g., 22, 23).

In order to understand *whether* attending the intervention was related to these observable differences among students, we layer quantitative methods in a staged fashion that allows the qualitative descriptions to remain central, while also integrating methods of quantification to compare across intervention and control groups, following Prowse and Camfield (23). Although novel in international development research, making qualitative methods of data collection a primary source of data in an evaluation should not be discouraged, as these methods are able to adhere to all of the “basic characteristics of randomized experiments” (23) (p. 56). Methods normally associated with qualitative research, such as FGDs (as employed in this study), are highly flexible: they can not only provide extensive process insights, as is common in qualitative methods, but can also be compared between arms of a study, and even quantized (transformed into quantitative data) in order to generate further insights.

Utilizing the flexibility of these data to investigate how observed skills and attitudes manifest and whether they differ across the arms has been central throughout the study, from the design of the FGD guide to the analysis. For instance, the design of the FGD guide in this study does not ask participants to recall their experiences, but rather, provides participants with a task and observes them completing it: participants were provided with a vignette and specific questions to probe how they interpreted it. In this manner, the FGD drew both from qualitative traditions, such as via including open ended questions and eliciting rich descriptions of interpretations and thought processes, as well as from quantitative traditions, such as laboratory or field experiments in behavioral economics and social psychology (e.g., 24, 25), that provide a task to participants and observe them completing it.

Quantizing data, which we undertake to allow additional interpretation, is common within mixed-methods research but should be done with care in order to avoid reducing qualitative data to numeric information (19). As such, we follow guidelines for quantizing qualitative data, including those established by Neale and colleagues (26), including that quantizing should only be done for “features that have been assessed for all the participants in a manner that allows for comparison” (p. 109). This study followed this criterion carefully, ensuring that the FGD guide was identical for intervention and control participants and was followed with fidelity. In addition, following Maxwell (19), we quantize the qualitative data in a manner that is “complementary to qualitative information rather than substituting for it” (p. 478).

### Setting

The study was conducted in Bihar, India, which is a state in north-eastern India, bordering Nepal. This state is home to over 120 million people (27) and has one of the lowest per-capita incomes in India: if Bihar were a country, it would sit between Eritrea and Liberia in terms of per-capita income (28). Women and girls are significantly disadvantaged in Bihar, and discrimination and gender-based violence are commonplace (29), causing girls to lag behind boys in a number of indicators, including their education and health (30).

### Participants

All schools that participated in the cRCT were rural, government-run schools in the Darbhanga and Patna districts of Bihar, had a student-teacher ratio of 50:1 or below, and were not implementing a similar resilience, life skills, or mental health promotion and prevention program in the middle school levels at the time of study launch.

A subsample of 12 schools that had participated in the cRCT were selected for this study: 8 schools that received Youth First (drawn from the cRCT intervention arms) and 4 that did not receive Youth First (drawn from the cRCT control arm). Schools were selected from two administrative blocks in Darbhanga district and two blocks in Patna district that had schools from each arm of the study. Within these blocks, schools were purposively selected to include only schools that were not closed due to COVID restrictions at the time of data collection in August 2021, where there were at least four teachers who had led Youth First program activities, and where at least four separate student groups had completed the Youth First program during the time of the intervention. These pragmatic inclusion criteria allowed the team to achieve the desired number of group discussions within resource constraints. In addition, the sample was selected such that it included schools with a variety of teacher quality ratings. These ratings had been made via observations during program implementation.

### Procedures

Students received 2 years of Youth First in 7th and 8th Standard (equivalent to United States 7th and 8th grade) from April 2018 to March 2020. Qualitative data were gathered from August to September 2021, which was approximately 3.5 years after the start of the intervention and 1.5 years after the completion of the intervention. In the intervening time between completion of Youth First and the qualitative data collection during this study, COVID-19 emerged, with multiple lockdowns that directly affected the study population and interrupted data collection, aborting the planned final round of quantitative data collection for the cRCT and delaying qualitative data collection for the current study by 6 months until we were able re-enter the field in August 2021.

Data were gathered by an external research agency that had not been involved in the intervention. This agency was based in New Delhi, India, and had experience conducting research in Bihar, India. Over its more than three decades of experience in India, the agency has conducted multiple multidisciplinary research projects. This agency collaborated on developing the tools for the study, oversaw and conducted data collection and entry, and provided a report based on initial thematic analysis.

All students from the cohort that had participated in the Youth First cRCT in each of the schools selected for the qualitative study were invited to participate in this study by CorStone program officers. Invitations were sent to students via contact information they had supplied at the start of the cRCT. Principals and teachers at the schools where the intervention had been held also helped to facilitate contact.

At each school, FGDs of eight to eleven students each were held separately for boys and girls. Parental consent was obtained from all students who volunteered to participate, and all students gave assent to participate by responding to a script read by FGD facilitators prior to the FGD. In total, 34 FGDs were conducted, including 26 with students from schools from the intervention arm and who participated in the YF program (15 all-girls discussions and 11 all-boys discussion) and 8 with students from schools from the control arm (4 all-girls discussions and 4 all-boys discussions).

Focus group discussion facilitators were employed by the external research agency. In addition to previous experience on other studies and other trainings they had received from the external agency prior to this study, they also received five full days of training on the concepts that they would be discussing during the FGDs, how to administer the FGD guide, and ethics. This team of facilitators also conducted mock FGD practice with a group of participants that were not included in the study. In each of the two districts (Patna and Darbhanga), there was a team of a senior manager that oversaw all activities and conducted quality assurance, as well as 6−7 FGD facilitators.

Focus group discussion facilitators recorded all discussions with consent and took written notes. Recordings from focus group discussions were transcribed in Hindi by researchers and all transcripts and field notes were then translated to English.

### Instruments

The study used two similar FGD guides to elicit students’ perspectives. FGD guides included one of two vignettes that described challenges frequently faced by students in the study area. One vignette was specifically for girls and presented only during the girls’ FGDs, and told the story of a fictitious girl in the community, Archana, facing pressures related to early marriage that could interfere with her education. The other vignette was specifically for boys and presented only during the boys’ FGDs, and told the story of a fictitious boy in the community, Ajay, facing pressures to work to help his family that could interfere with his education. These vignettes were collaboratively developed by local CorStone staff and the external research agency such that they were relevant to youth in the area and were likely to elicit inter- and intra-personal psychosocial skills that are targeted via Youth First as proximal outcomes. See Table 1 for the English translations of the girl and boy vignettes.

**TABLE 1:** English translations of vignettes used for study focus group discussions.

Vignette for girls’ discussions	Vignette for boys’ discussions
Archana is a young girl in this area who was dreaming of becoming a doctor to help people. She was not only dreaming but also working hard in middle school to score good marks, making progress in school toward achieving that dream. Last year when schools closed, Archana kept her motivation to continue her studies. It was especially difficult because her family does not have a smartphone. She and her siblings had to share when they could borrow one from a close neighbor. During the second wave of COVID-19, her father began speaking of arranging her marriage to an older man in the next village, who does not approve of too much education for girls. Archana’s mother is not disputing the idea, and now it seems to be settled. Archana cannot eat or sleep. She is only 15 years old and does not want to lose her dream but does not know what to do.	Ajay is studying in Standard 8 and looking forward to high school. He lives with his mother and two younger siblings. His father is in Delhi where he was working as a cook, sending money each month to help with family expenses. Since lockdowns happened, his father lost that job. He stayed in Delhi but has not gotten regular work since then. Ajay noticed lately his father calls his mother more often. She is often very upset after these calls. His mother starts to get angry for no reason and beats his siblings. His mother also decided Ajay has to quit school, drop out, and get a job. He feels helpless and is always shouting at everyone. Ajay cannot eat or sleep. He is only 15 years old, wants to study and wants everyone to stop fighting, but does not know what to do.

The FGD guides sought to understand students’ perspectives and skills with respect to these vignettes. They specifically probed Youth First’s hypothesized proximal outcome areas, including problem identification and logical thinking, problem solving and coping mechanisms, empathy, identifying internal assets and skills, using social relationships to manage difficulties, and attitudes related to gender.

In contrast to the way in which many qualitative FGD guides are constructed, the guides used in this study did not ask explicitly about students’ time in Youth First, nor about their memories nor thoughts about it. Rather, the guides were constructed as an experiment that was presented in the same way to participants in both intervention and control arms. In each FGD, the vignette was presented, then a single set of questions was asked for participants to explain and reflect on what they saw and thought about the vignettes. The vignette and set of questions did not differ between intervention and control groups of the same gender. The study also gathered student demographics at the same time point as the FGDs.

### Analysis

Analysis consisted of an iterative thematic analysis process (drawn from qualitative techniques), followed by a quantizing process (to transform data into quantitative data for further insights and analysis, drawn from quantitative techniques). First, the external research agency examined FGD transcripts against broad *a priori* themes that mirrored areas of probing in the FGD guides: (i) problem identification and logical thinking; (ii) problem solving and coping mechanisms; (iii) empathy; (iv) internal assets and skills; (v) relationships and support; and (vi) gender. The agency summarized responses of students during FGDs within these themes, separated by respondent characteristics (i.e., intervention, control, girls, and boys), and tabulated these summaries in an Excel spreadsheet. The spreadsheet was then examined and sub-themes emerged inductively. Table 2 provides the broad *a priori* themes established at outset and subsequent sub-themes that emerged inductively during analysis.

**TABLE 2:** Themes and sub-themes used during analysis.

Themes (established deductively)	Sub-themes (emerged inductively)
Problem identification and logical thinking	Problems and challenges identified
	Problems and challenges from others’ perspectives considered
	Root causes of the problem(s) identified
Problem solving and coping mechanisms	Potential solutions offered
	Possible resolutions for the story suggested
Empathy and prosocial behaviors	Ways to help a friend facing similar challenge articulated
Internal assets and skills	Internal assets that could help in the situation identified
	Skills and talents that could help in the situation identified
Relationships and support	Ways that relationships could help in the situation identified
Gender	What would change if main character was a different gender articulated

The external research agency then provided a report of findings to other research team members who had in-depth familiarity of the intervention and its targets. The first author (KL) examined this report and identified any psychosocial assets, skills, or attitudes that would fall into the broad category of proximal outcomes of the intervention (inter- and intra-personal psychosocial assets, skills, and attitudes). These assets, skills and attitudes were then compared across study arms in terms of differences or similarities in number, level, or quality of responses.

Differences were categorized as: intervention responded similarly to controls, controls responded more favorably than intervention, or intervention responded more favorably than controls. The second author (PC) then examined the summary from the research agency and performed the same categorizations, blind to the study arm of the participants and blind to the categorizations made by the first author. Categorizations of the first and second author were then compared. Initial agreement between the first and second author was 98%. All discrepancies were discussed and the wording of one of the outcomes was changed slightly. Discrepancies were then resolved with 100% agreement.

Observed outcomes were then tabulated according to these three categories (intervention fared similarly to controls, controls fared better than intervention, and intervention fared better than controls). Finally, higher-order constructs were generated, grouping observed outcomes for ease of interpretation. This component of the analysis contributed to understanding *how* participants exhibited inter- and intra-personal psychosocial assets and skills after the intervention, in comparison to those who did not attend the intervention.

In order to quantize the information for further quantitative interpretation, allowing us to answer the question of *whether* attending the intervention was associated with these observed differences, the categories of “intervention responded similarly to controls,” “controls responded more favorably than intervention,” and “intervention responded more favorably than controls” were dummy coded as 0, −1, and 1, respectively. Then, means of these dummy codes were computed for the observed outcomes within boys’ FGDs and separately for the observed outcomes within girls’ FGDs. This dummy coding process allowed us to investigate whether participating in the intervention was associated with higher levels of skills vs. not participating in the intervention. In this method, if the mean of the coded outcomes is positive (greater than 0), this indicates that those who attended the intervention exhibited higher skill levels than those who did not attend. If the mean is negative (less than 0), this indicates that those who attended the intervention exhibited lower skill levels than those who did not attend. If the mean is zero, this indicates that those who attended the intervention and those who did not attend exhibited similar skill levels.

We hypothesized that the mean of the coded outcomes would be greater than zero, indicating that those who attended the intervention exhibited higher skill levels than those who did not attend. To test this hypothesis, we compared the observed mean for the boys’ FGDs and for the girls’ FGDs to zero, using a single-sample one-tailed *t*-test. The threshold for statistical significance was set at *p* < 0.05.

## Findings

### Description of sample

A profile of the student sample appears in Table 3. Across the intervention and control groups, 42% of students participating in the focus group discussion were boys and 58% were girls. Fifty-nine percent of students were in Darbhanga schools while 41% were in Patna schools. Ninety-three percent of students were enrolled in 10th Standard at the time of the study.

**TABLE 3:** Demographic profile of the sample.

	Boys		Girls		Overall	
			
	Count	%	Count	%	Count	%
Total	136	100%	186	100%	322	100%
**District**						
Darbhanga	81	60%	110	59%	191	59%
Patna	55	40%	76	41%	131	41%
**Standard**						
8th	2	1%	1	1%	3	1%
9th	5	4%	5	3%	10	3%
10th	129	95%	169	91%	298	93%
**Education aspirations**						
Secondary school^1^	4	3%	6	3%	10	3%
Senior secondary school^2^	35	26%	66	35%	101	31%
Diploma^3^	20	15%	13	7%	33	10%
Graduation^4^	65	48%	79	42%	144	45%
Master’s degree	9	7%	7	4%	16	5%
Doctorate	1	1%	6	3%	7	2%
Polytechnic degree	1	1%	8	4%	9	3%
**Marital status**						
Married	0	0%	3	2%	3	1%
Unmarried	136	100%	183	98%	319	99%

^1^Secondary school = 9th and 10th standard.

^2^Senior secondary school = 11th and 12th standard.

^3^Graduation = degree conferred with three years of study after 12th standard.

^4^Diploma = degree provided with between 3 months and 2 years of study, which can be undertaken at any time after completing 10th standard.

Table 4 summarizes the sub-themes identified and corresponding outcomes that arose within the focus group discussions. It also summarizes which arms (intervention or control) fared better, by gender.

**TABLE 4:** Intervention vs. controls by outcomes arising from focus groups.

Gender	Sub-themes	Outcomes sought by intervention	Intervention (I) vs. Controls (C)
Girls	Problems and challenges identified	Awareness that the situation is “wrong,” is a problem, and is illegal	I∼C
		Number of problems identified	I > C
	Problems and challenges from others’ perspectives considered	Number of problems identified from each person’s perspective in the situation	I > C
		Awareness of internal/emotional concerns from each person’s perspective	I > C
	Root causes of the problem(s) identified	Awareness that the situation is linked to social, structural norms/inequities	I∼C
		Number of root causes identified	I > C
		Awareness that situation/system interferes with self-expression of main character	I > C
	Potential solutions offered	Desire for main character to take concrete and strong steps to solve the problem	I∼C
		Identified that reporting this illegal practice is possible	I∼C
		Number of solutions identified	I > C
		Creativity and innovation of solutions suggested	I > C
		Identified assertive communication as a solution	I > C
		Identified 1098 helpline as a potential resource	I > C
		Identified teachers as a potential resource	I > C
		Identified importance of main character’s emotional awareness/management/communication	I > C
	Possible resolutions for the story suggested	Agreed that it is likely for most girls that there would be a “bad ending”	I∼C
		Identified educational, career, and responsibility consequences of a “bad ending”	I∼C
		Identified emotional and physical health consequences of a “bad ending”	I > C
		Detail in vision of what a “good ending” would look like	I > C
	Ways to help a friend facing similar challenge articulated	Related to the situation and wanted to help	I∼C
		Identified providing emotional support as a way to help	I∼C
		Identified talking to her parents as a way to help	I∼C
		Identified encouraging her to reach her goals as a way to help	I∼C
		Identified giving material resources (notebook/smartphone) as a way to help	I∼C
		Number of ways to help proposed	I > C
		Creativity and innovation of ways to help that were proposed	I > C
		Identified getting other adults involved (parents, teachers, police) as way to help	I > C
		Identified telling her about 1098 helpline as a way to help	I > C
	Internal assets that could help in the situation identified	Number of internal assets identified	I > C
		Ability to identify *how* specific internal assets would help in the situation	I > C
	Skills and talents that could help in the situation identified	Number of talents/skills identified	I > C
	What would change if the main character was a different gender articulated	Awareness that situation would have a more likely positive ending for a boy	I∼C
		Awareness that this inequality is wrong and should change	I∼C
Boys	Problems and challenges identified	Awareness that the situation is “wrong” and is a problem	I∼C
		Number of problems identified	I > C
	Problems and challenges from others’ perspectives considered	Number of problems identified from each person’s perspective in the situation	I∼C
	Root causes of the problem(s) identified	Awareness that the situation is linked to social, structural norms/inequities	I∼C
		Number of root causes identified	I > C
	Potential solutions offered	Desire for main character to take concrete + strong steps to face + solve the problem	I∼C
		Identified assertive communication as a solution	I∼C
		Identified working together with friends as a solution	I∼C
		Identified relevant ways to make money in the situation	I∼C
		Creativity and innovation of solutions identified	I > C
		Identified managing emotions as a solution	I > C
		Identified drawing from internal strengths + resources as a solution	I > C
		Identified sharing goal with others as a solution	I > C
	Possible resolutions for the story suggested	Agreed that it is likely for most boys that there would be a “good ending”	I∼C
		Identified educational, career, and responsibility consequences of a “bad ending”	I∼C
		Identified emotional consequences of a “bad ending” *and* a “good ending”	I > C
		Identified how strengths and skills will help in the situation	I > C
		Number of possibilities identified of how the situation could “end”	I > C
	Ways to help a friend facing similar challenge articulated	Related to the situation and wanted to help	I∼C
		Identified giving material resources (notebook/financial) as a way to help	I∼C
		Identified providing emotional support as a way to help	I > C
		Identified helping to care for his physical health as a way to help (make sure has food)	I > C
		Identified encouraging him to reach his goals as a way to help	I > C
		Identified getting other adults involved (parents, teachers, family) as way to help	I > C
		Identified helping him come up with solutions as a way to help	I > C
		Number of ways to help proposed	I > C
		Creativity and innovation of ways to help that were proposed	I > C
	Internal assets that could help in the situation identified	Number of internal assets identified	I > C
		Number of internal assets identified	I > C
	Skills and talents that could help in the situation identified	Number of talents/skills identified	I > C
	What would change if the main character was a different gender articulated	Awareness that situation would have a more likely negative ending for a girl	I∼C
		Awareness that this inequality is wrong and should change	I∼C
		Detail provided about what the situation would be like for a girl	I > C

I, Intervention; C, Controls; I∼C, Intervention and controls fared similarly; I > C, Intervention fared better than controls. There were no observed cases in which controls fared better than intervention.

### Similarities and differences between intervention and control arms

Thirty-three outcomes arose within girls’ and boys’ focus group discussions, respectively. Thirteen of these for each gender (39 percent) were cases in which intervention and control arms fared similarly; for the remaining 20 outcomes identified for each gender (61 percent), girls or boys within the intervention arm fared better than those in the control arm. There were no cases in which controls fared better than the intervention for either gender.

Table 5 provides the commonalities observed across the arms for girls and boys, and Table 6 provides the differences observed across the arms. These tables also provide the higher-order constructs that were generated to summarize the observed outcomes.

**TABLE 5:** Outcomes observed to be similar across the arms.

Higher-order constructs	Outcomes sought by intervention	Similar across intervention and control in girls’ FGDs	Similar across intervention and control in boys’ FGDs
Awareness that situation is linked to social inequities	Awareness that the situation is linked to social, structural norms/inequities	✓	✓
	Awareness that situation would have a more likely positive ending for a boy	✓	✓
	Believed that it is likely for most girls in their community that there would be a “bad ending”	✓	
	Believed that it is likely for most boys in their community that there would be a “good ending”		✓
Awareness that the situation is “wrong”	Awareness that this inequality is wrong and should change	✓	✓
	Awareness that the situation is “wrong” and is a problem	✓	✓
	Desire for main character to take concrete and strong steps to solve the problem	✓	✓
	Identified educational, career, and responsibility consequences of a “bad ending”	✓	✓
	Identified that this practice is illegal and reporting it is possible	✓	
Related to situation and had basic ideas of how to support	Related to the situation and wanted to help	✓	✓
	Identified giving material resources as a way to help	✓	✓
	Identified providing emotional support as a way to help	✓	
	Identified talking to her parents as a way to help	✓	
	Identified encouraging her to reach her goals as a way to help	✓	
	Identified assertive communication as a solution		✓
	Identified working together with friends as a solution		✓
	Identified relevant ways to make money in the situation		✓
Basic perspective-taking	Number of problems identified from each person’s perspective in the situation		✓

**TABLE 6:** Outcomes in which intervention arms were observed to fare better than control arm.

Higher-order constructs	Outcomes sought by intervention	Girls in intervention fared better than control in FGDs	Boys in intervention fared better than control in FGDs
Nuanced and multifaceted awareness of the problem	Number of problems identified^1^	✓	✓
	Number of root causes identified^1^	✓	✓
	Awareness that situation/system interferes with self-expression of main character^2^	✓	
Empathic awareness of others’ perspectives and emotions	Number of problems identified from each person’s perspective in the situation^1^	✓	
	Awareness of internal/emotional concerns from each person’s perspective^3^	✓	
Creative, innovative, assertive, self-aware, and goal-directed problem-solving	Number of solutions identified^1^	✓	
	Creativity and innovation of solutions suggested^3^	✓	✓
	Identified assertive communication as a solution^2^	✓	
	Identified 1098 helpline as a potential resource^2^	✓	
	Identified teachers as a potential resource^2^	✓	
	Identified importance of main character’s emotional awareness/management/communication^2^	✓	
	Identified managing emotions as a solution^2^		✓
	Identified drawing from internal strengths as a solution^2^		✓
	Identified sharing a goal with others as a solution^2^		✓
Creative, innovative, collective, and empathic helping approach	Number of ways to help proposed^1^	✓	✓
	Creativity and innovation of ways to help proposed^3^	✓	✓
	Identified getting other adults involved (other parents, teachers, police) as way to help^2^	✓	✓
	Identified telling about 1098 helpline as a way to help^2^	✓	
	Identified providing emotional support as a way to help^2^		✓
	Identified helping to care for physical health as a way to help^2^		✓
	Identified encouraging to reach his goals as a way to help^2^		✓
	Identified helping him come up with solutions as a way to help^2^		✓
In-depth awareness of strengths, resources and skills to draw upon	Number of internal assets identified^1^	✓	✓
	Number of talents/skills identified^1^	✓	✓
	Ability to identify *how* specific strengths would help in the situation^3^	✓	✓
Detailed vision of future paths and their ramifications	Detail in vision of what a “good ending” would look like^3^	✓	
	Number of possibilities identified of how the situation could “end”^1^		✓
	Identified physical health consequences of a “bad ending”^2^	✓	
	Identified emotional consequences of a “bad ending”^2^	✓	✓
	Identified emotional consequences of a “good ending”^2^		✓
Nuanced understanding of gender inequities	Detail provided about what the situation would be like for a girl^3^		✓

^1^Greater number in the intervention arms than in the control arm.

^2^Present in the intervention arms and not observed at all in the control arm.

^3^More advanced level of this skill observed in intervention arms than in control arm.

#### Commonalities observed across arms

Participants in the intervention and control arms responded similarly to one another in a number of ways (see Table 5), including in their awareness that the situation in the vignette is linked to social inequalities, their awareness that the situation is “wrong,” and their empathy for the main character and basic ideas of how to support her or him.

##### Awareness that the situation is linked to social inequalities

In terms of their awareness that the situation is linked to social inequalities, girls and boys from both the intervention and control arms explained what was going on in the vignette similarly, and they felt that the situation described in each of their respective vignettes was common in their area. Girls in both intervention and control arms were aware that social inequalities between boys and girls existed, and they attributed the situation in the vignette to the existence of these unequal social norms. For instance, a girl in the intervention arm noted that “most of the elderly people and neighbors say that [when] the daughter has grown up, marry the daughter off rather than sending her to school.” A girl in the control arm similarly noted, “the rules and customs of the society in which they live had to be followed.” Along similar lines, boys in both arms attributed the situation in the vignette to poverty, which is the specific instance of social inequality that is highlighted in the boys’ vignette.

Boys and girls across both arms identified that the vignettes would be likely to have a more positive outcome if the main character was a boy vs. a girl. Girls believed that for most girls in their community, there would likely be a “bad” ending to the vignette they were discussing; boys believed that for most boys in their community, there would likely be a “good” ending to the vignette they were discussing.

##### Awareness that the situation is “wrong”

Girls and boys from both arms were also clear to a similar extent not only that the situation was “wrong,” but also that the main character should do something concrete to change it. They also believed that the inequality that had caused the situation itself was unfair and should change. For instance, girls in the intervention group suggested that the girl in the story should advocate for her rights. Girls in the control group similarly suggested that the girl should try to find a way to avoid getting married now, and that by doing so she could inspire others in the society so that the practice of early marriage will end.

In addition, participants in focus groups of both arms and both genders were able to articulate the consequences that would await the main character if the situation did not end well regarding education, career, and responsibilities. For example, girls across intervention and control groups imagined that Archana is likely to get married, drop out of school, become a housewife, and eventually a mother. As one intervention group girl said, 2 or 3 years later, “Archana would have become a mother and she would also have the responsibility of children.”

There was also a similar level of awareness among girls in the intervention and control arms that the practice of early marriage is illegal and that it is possible to report it, as evidenced by suggestions from girls across both arms that the girl in the vignette could report the situation to the police. For instance, one control arm girl said that the girl in the vignette should get “help from the police, because if police will tell her parents that child marriage is illegal then her parents will get convinced.” An intervention group girl said, “If Archana had taken the help of the police, her parents would have been scared and would not fix her marriage.”

##### Related to the situation and had basic ideas of how to support

Boys and girls in both arms had in common that they all related to the situation and had some basic ideas of how they would help support someone who was in the situation of the main character in the vignette. As one intervention arm girl said, “Me and Archana belong to the same community, so I can understand her feelings.” Girls and boys from both arms also mentioned giving material resources as a way to help the main character. Beyond this, the ways in which boys and girls suggested that they could help the main character differed.

Girls across both intervention and control arms both indicated that if they knew someone in this situation, they would provide emotional support, talk to the girl’s parents, and provide encouragement to the girl to reach her goals. For instance, one intervention arm girl said, “I would encourage her, [by saying] please don’t give up! Let’s talk to your parents.” By contrast, as described in the section titled “Creative, innovative, collective, and empathic helping approach,” below, boys in the intervention arms identified providing emotional support and encouragement to the main character to reach his goals, but boys in the control group did not mention these as potential ways they could support the main character.

Boys in both intervention and control groups identified assertive communication as a solution to the problem in the vignette, suggested working together with friends, and also identified relevant ways to make money in the situation, to a similar extent across both arms. As one boy in the intervention group mentioned, Ajay “could help the needs of himself and his family by working a part-time job.” Similarly, a boy in the control group mentioned, “Ajay should do small work along with his studies. This reduces his problem.” By contrast, as described in the section titled “Creative, innovative, assertive, self-aware, and goal-directed problem-solving,” below, girls in the intervention arms identified assertive communication as a solution, though girls in the control arm did not. Girls also did not identify working together with friends or relevant ways to make money as ways to help the main character in the vignette in either arm, though these solutions may have been less likely to have been relevant to the vignette with which the girls were presented.

##### Basic perspective-taking (commonality observed among boys only)

Boys in the intervention and control arms were able to identify a similar number of problems from each person’s perspective in the situation. For instance, boys from both arms identified that from the father’s perspective, the major problem is that he lost his job, that from the siblings’ perspective, the major problem is that the mother frequently scolds and beats them, and that the economic concerns are problems for everyone in the family. As one intervention arm boy said, “His parents [and] his siblings are facing problems because they don’t have money.”

As described in the section titled “Empathic awareness of others’ perspectives and emotions,” below, girls in the intervention arms showed greater perspective-taking abilities than girls in the control arm. Perspective-taking abilities of girls (in all arms) were also greater than those of boys (in all arms), as indicated by the number of problems they were able to identify from each person’s perspective and the depth of description of these problems.

#### Differences observed across arms

As presented in Table 6, there were many differences observed between the intervention and control arms, all of which favored the intervention arm. In particular, those in the intervention arms brought a higher-order set of skills and assets to their interpretation of the vignette compared to the students from the control schools. Students from the intervention arm had a more nuanced and multifaceted awareness of the issues in the situation; had a more empathic awareness of others’ perspectives and emotions; suggested more creative, innovative, self-aware, goal-directed, and assertive problem-solving strategies; suggested more creative, innovative, collective and empathic helping approaches; had a more in-depth awareness of strengths, resources, and skills that could be drawn upon in the situation; held a more detailed vision of potential future paths beyond the situation, both good and bad; and boys in particular had a more nuanced understanding of gender inequalities compared with those in the control arm.

##### Nuanced and multifaceted understanding of the problem

Girls and boys in the intervention arms were better able than those in the control arm to identify what the problems were in the situation and had a more nuanced view of what the underlying causes of the problems were. They identified more root causes of the problems, including both structural problems and how these structural problems affected the main character in the vignette as an individual. The potential root causes that girls in the intervention group listed for the situation were numerous and widely varied in contrast to the control group. For example, girls in the control group mentioned that poverty, COVID-19 lockdowns, illiterate parents who do not value education, and the fact that in society girls are not allowed to study and go out could have all been root causes for the situation presented in the vignette. Girls in the intervention group mentioned all of these root causes as well, but also mentioned additional root causes of the situation, including but not limited to that the following may have played a role: the fact that the main character could not express herself, that girls are considered as a “liability in society,” that the girl’s age might make it hard for her to think of what to do in the situation, that the existence of dowry as a practice contributes to the desire to marry girls early, and that there is discrimination between girls and boys in society. As one girl in the intervention arm said, “If the dowry system is stopped, then every boy and girl will be able to study.” Another intervention arm girl stated, “Girls are not given liberty. That’s what this problem is connected to”.

##### Empathic awareness of others’ perspectives and emotions (girls only)

Although boys showed roughly the same level of basic perspective-taking across the intervention and control arms [see the section titled “Basic perspective-taking (commonality observed among boys only),” above], girls in the intervention arms showed a more empathic and advanced perspective-taking ability than those in the control arm (though, as mentioned previously, both intervention and control girls were more empathic and advanced in terms of perspective-taking than boys).

Girls in the intervention arms showed a greater ability to understand others’ perspectives and were able to articulate emotional concerns clearly from the perspective of each person in the situation. Girls in the control arm did not articulate as many different problems from others’ perspectives, nor did they identify internal or emotional concerns of different people in the vignette to the same extent as girls in the intervention arms volunteered. For instance, girls in the intervention arm identified that the parents in the vignette faced many issues, including the pressure to follow social norms and fulfill their responsibilities by marrying their daughter early, the prospect of taunts from other villagers if they do not get her married, and potential concerns about finances. Girls in the control group did not identify any of these concerns from her parents’ perspectives.

##### Creative, innovative, assertive, self-aware, and goal-directed problem-solving

Problem solving strategies that were suggested were also markedly different across arms, particularly for girls. Girls in the intervention arms suggested many more solutions to the problems in the vignette than those in the control arm did. Although boys in the intervention arms didn’t come up with markedly more solutions to the problem than those in the control arm, the creativity and innovation of their solutions vs. the control group was clear.

Both girls and boys in the intervention arms also identified several solutions to the problem in the vignette that those in the control groups did not mention at all. The nature of these suggested solutions was different for girls vs. boys in the intervention arms. Girls in the intervention arms identified assertive communication, the existence of a government helpline (the “1098 helpline”) to report child marriage, the idea that teachers were a potential resource who could help, and the importance of becoming more aware of emotions and communicating them to others better. Exemplifying the belief in girls’ power through assertive communication, one intervention group girl stated, “We should trust ourselves because if we have trust in ourselves then we will raise our voices.” Another said, “If Archana had had the courage to speak, she could have.” The girls in the control arm did not mention any of these as potential solutions.

Boys in the intervention arms identified that the boy could manage his emotions, draw from internal strengths, and share about his goals with others as potential solutions. The boys in the control arm did not mention any of these as potential solutions. As one intervention arm boy said, “[Ajay could have used] perseverance – if he had been experienced to face with complex situations, then, he could have easily come out of this problem.” Another said, “If I have character strengths, I can deal with it if there is any problem at home [like Ajay is facing].”

##### Creative, innovative, collective, and empathic helping approach

Girls and boys in the intervention and control arms suggested different ways that they could help the main character in the vignette, though the exact content of the suggestions differed by gender. Girls and boys suggested a greater number of ways to help the main character in the intervention arm vs. the control arm, and they also showed more creativity and innovation in what they suggested. Both girls and boys in the intervention arms suggested getting other adults involved as a potential way to help, including other parents, teachers, and police, drawing on a support system of adults that went well beyond the adults in the story. Those in the control arm did not suggest reaching out to other adults as a possibility, with the exception of neighbors.

Other ways girls and boys in the intervention arms suggested that they could help the main character differed by gender. Girls in the intervention arms identified directing the girl to the 1098 helpline as a way to help her, whereas girls in the control arm did not mention this possibility. Boys in the intervention arms suggested providing emotional support to the main character, providing encouragement to the main character to reach his goals, and helping him come up with and brainstorm solutions. Boys in the control arm did not suggest any of these strategies.

##### In-depth awareness of strengths, resources, and skills to draw upon

Both girls and boys in the intervention arms were also highly aware of what kinds of strengths, resources and/or skills that the main character could draw upon, and were able to explain how these strengths, resources, or skills would be helpful. Those in the control arm identified fewer internal assets/strengths and fewer talents and skills, and they were also not able to provide nearly as many examples nor the same level of clarity as those in the intervention arms regarding how the strengths or resources would be helpful in the situation.

For example, boys in the control arm only identified being hardworking and the ability to understand the problem as potential internal assets that could help the main character in the situation. Boys in the intervention arm identified not only being hardworking but also self-confidence, enthusiasm, intelligence, honesty, remaining calm, not being overly trusting of unknown people, patience, thinking optimistically, sympathy, and ability to make a thoughtful decision, among other potentially helpful assets that they identified.

As one girl in the intervention arm described, “Persistence, zest, and self-regulation [are important]. [Also] intelligence will help her to not give up, and she will be able to manage her fear.” Another girl in a different intervention FGD said, “Self-confidence, patience and bravery – these strengths will help us to overcome any kind of situation. We can convince our parents with self-confidence.”

##### Detailed vision of future paths and their ramifications

Compared to girls and boys in the control arm, girls and boys in the intervention arm showed a more detailed vision of what the future could look like for the main character in the story in terms of what they believed would be a “good” and a “bad” ending for the main character. In particular, girls in the intervention arms provided greater detail than those in the control arm regarding what they thought a “good” ending would look like for the girl. Girls in the control arm only described the potential paths the girl in the vignette might take in terms of educational and responsibility ramifications. However, girls in the intervention arms provided not only these educational and responsibility ramifications, but also a more robust picture of what would happen emotionally for the girl in the case of a “bad” ending and what would happen in terms of her physical health if she got married early. Girls in the intervention group mentioned that she might be “depressed” and “upset” if she does have to get married early in the future, whereas the girls in the control group did not mention any emotional ramifications of getting married early beyond a few girls who mentioned she might feel “bad” or “sad.” As one girl in the intervention group put it, “If she gets married, her dream of becoming a doctor will be shattered, due to which she will stay stressed.” Another girl in the intervention arm described that if Archana gets married, “she will try to live alone [keep to herself], she won’t talk much, she won’t eat properly, and she will feel stressed and suffocating.”

Boys in the intervention arms identified more possible ways that the situation in the vignette could end or progress than boys in the control arm. In the intervention arm, boys predicted that the main character might complete his studies, help solve his parents’ problems, complete his studies, and get married. They mentioned that he might also help his siblings receive an education. Boys in the control arm only identified short-term ways that the situation could end or progress, including that the father and/or that the main character could find a job, and then lockdown would lift so that the main character would start studying again. Boys in the intervention arms also identified what would happen for the boy emotionally for what they believed would be a “bad” or a “good” ending, mentioning that the main character is likely to feel “concerned” before the situation is resolved, then feel “happy” when and if he is able to return to school. Boys in the intervention arm readily put themselves in Ajay’s place and described what their emotions would be as the situation progressed. For example, one intervention group boy said, “When the goal is not accomplished, I myself [would] feel sad and anxious, which makes me feel angry and its effect will be bad on others, too.” Boys in the control arm did not mention any emotional consequences that they believed would happen as the vignette proceeded or ended.

##### Nuanced understanding of gender inequalities (differences observed among boys only)

Compared with boys in the control arm, boys in the intervention arm were able to provide more detail about what the situation would be like for a girl. For example, boys in the intervention arm mentioned that parents get influenced by other parents and want to marry off their daughter, and that if the situation were inverted and a girl went out to try to work to help her family, society would call her “bad” and say that it was “wrong” for a girl to work outside the home. Boys in the control arm only mentioned that a girl in the situation of the boys’ vignette would likely work from home and do a different kind of work than if she were a boy. This difference was not seen in the girls’ focus groups, in which both intervention and control girls were able to provide a similar level of detail regarding what the situation in the vignette would be like for a girl vs. a boy.

### Results of statistical analysis of quantized data

Once the observed outcomes were dummy coded (1 = intervention fared better than controls, 0 = intervention fared similarly to controls, and −1 = controls fared better than intervention), the mean (M) of the outcomes was 0.61 (*SD* = 0.50; this was the same for boys’ and girls’ FGDs). This mean reflected that 61% of the outcomes represented instances in which the intervention fared better than controls, 39% of the outcomes represented instances in which the intervention fared similarly to controls, and 0% of the outcomes represented instances in which the controls fared better than the intervention.

This mean was significantly different from 0 or any negative number, which is the mean that would have been expected if those who received the intervention provided similar or lower level responses vs. those who did not receive the intervention, *t*(32) = 7.02, *p* < 0.001. Table 7 provides *t*-test results.

**TABLE 7:** *T*-test results comparing observed outcomes to null hypothesis.

	Single-sample one-tailed *t*-test*					
	
	*N*	M**	*SD*	*df*	*t*	*p*
Outcomes observed in girls’ FGDs	33	0.61	0.50	32	7.02	<0.001
Outcomes observed in boys’ FGDs	33	0.61	0.50	32	7.02	<0.001

N, number of observed outcomes; M, mean; SD, standard deviation; *df*, degrees of freedom; *t*, t statistic; p, p-value. *T-test compares observed M to null hypothesis of *M* ≤ 0. **Mean of outcomes observed in FGDs, coded as 1, intervention fared better than controls; 0, intervention fared similarly to controls; −1, controls fared better than intervention.

## Discussion

This study sought to understand the differences in intra- and inter-personal psychosocial assets, skills, and attitudes displayed by participants of a resilience-based intervention, Youth First, vs. non-participants. It intended to understand, within a broad set of proximal outcomes of interest, what assets, skills, and attitudes arose following this intervention.

### Observed differences between Youth First participants vs. non-participants

The majority of assets, skills, and attitudes identified in the study, approximately 60 percent, reflected higher-order skills or understanding in respondents from the intervention arm vs. the control arm. The remaining assets, skills, and attitudes observed, approximately 40 percent, were similar across students in both arms. There were no instances in which the control arm students exhibited better skills or understanding than the intervention arm. Quantitative analyses revealed that this difference was unlikely to be due to chance.

Out of these findings, two portraits of participants emerged: one portrait of youth in the control arm, and one portrait of youth in the intervention arm. Students in the control arm showed a basic level of understanding of social inequalities, a desire to do something about situations that they believed to be morally wrong, a desire to help friends in difficult situations, and a number of basic ideas of how they could provide support to others in challenging situations, including material resources and working together. By contrast, students in the intervention arm showed all of these and more: their responses reflected not only these ideas and skills, but also nuanced, creative, complex, and higher-order thinking. Students in the intervention arm had a multifaceted view of issues that youth faced in their areas; had an empathic awareness of others’ concerns and an ability to see complex issues from many others’ perspectives; came up with creative, innovative, self-aware, assertive, goal-directed, and empathic ideas of how to solve (and/or help others solve) difficult situations; were highly aware of a multitude of strengths, skills and resources that they or others could draw on to resolve difficult situations; had a detailed vision of what future paths could exist for girls and boys in their area who face problems related to gender inequalities and/or poverty; and held a nuanced understanding of the experience of gender inequalities from multiple perspectives.

These findings suggest that psychosocial skills and assets in youths are modifiable and can be strengthened through mental health promotion and prevention interventions in schools in LMICs, supporting previous findings regarding Youth First (16, 17) and similar programs (12). In addition, it is particularly encouraging that these differences were apparent 1.5 years after the intervention ended (3.5 years after the intervention began). Long-term follow-ups are rare among mental health promotion and prevention programs for LMIC youth, and few have followed-up with participants after more than a few months post-intervention (12). This study suggests that it is possible to clearly observe differences among those who have participated in such interventions vs. those who did not even 1.5 years after intervention.

In addition, findings suggest that Youth First may function in part by building on basic skills and assets of youths and helping them to amplify these strengths, adding nuance, complexity, and higher-level skills and attitudes. The fact that Youth First participants exhibited these high-level skills and attitudes is particularly encouraging when considering how to build programs for youth in LMIC settings that enable them to meet daily complex challenges. In particular, as youth encounter challenges and seek ways to thrive — particularly when the challenges are related to gender, poverty and/or other entrenched social inequalities — higher-order thinking and nuance in skills are crucial (6). Further, as Youth First emphasizes a strength- or asset-based approach (e.g., 31, 32), in which one of its main pedagogies includes helping youth identify and amplify what is already “good,” “strong,” and/or “going well” for them, this finding supports that this pedagogy may mirror a mechanism that occurs within students’ experiences.

### Implications for Youth First adaptations and application at scale

The version of Youth First implemented during this study has evolved from an earlier version of the program that had been found to be effective in a previous randomized controlled trial (at the time, it was called Girls First) (16, 17). Although the program content in the current study was similar to the program implemented during the previous trial, this version of the program reduced the number of sessions per year, expanded the program to include all genders rather than just girls, and trained government schoolteachers to serve as facilitators rather than community women. As government schoolteachers represent a large existing workforce who can be reasonably trained and overseen to conduct this program among groups of students, transitioning to implementation by schoolteachers represents an important evolution of the program’s scalability and potential for institutionalization. Although these changes were important for scalability and institutionalization reasons, they also resulted in a program that was less intensive, less tightly controlled and observed, using service providers endogenous to existing systems, and with a more heterogeneous population than in the previous trial. It is a frequently observed phenomenon in other trials that these types of changes can lead to eroding or even disappearing effects (33).

It is therefore promising that many observable differences emerged related to the intended proximal effects of Youth First (inter- and intra-personal psychosocial assets and skills) between the intervention and control group. These findings suggest that Youth First may be capable of producing effects even in its more scalable version. This version could be easily adopted and replicated within government programming in the future, as it aligns with the resources available to the Bihar government school system, as well as those available within many other LMIC government school systems.

### Defining outcomes in future low- and middle-income countries studies of preventive and promotive mental health interventions

One of the strengths of this study was that it provided details of skills, assets, and attitudes that Youth First participants exhibited without limiting the study’s scope to a small set of strictly predefined outcomes. By taking this approach, a list of outcomes was generated that can be useful to future researchers. Examining the categories that emerged from the data, two main implications are evident, each of which has relevance beyond this study to future studies that aim to quantify outcomes: first, the outcome categories identified do not map clearly to categories established via previous studies; and second, basic skills may be present in youths even without intervention, so higher-level skills are important areas in which to explore change. Each of these implications is elaborated below.

#### Outcomes identified do not map clearly to categories established via previous studies

Although many outcomes emerged in this study that fit the broad definition of inter- and intra-personal psychosocial skills and attitudes (Youth First’s proximal targets), the categories do not map strictly to widely used definitions of such outcomes, or even to measures that already exist. In fact, most observed outcomes combine multiple constructs. For instance, to our knowledge, there is no measure that has been developed in any population that specifically measures “Creative, innovative, assertive, self-aware, and goal-directed problem-solving.” However, this described the type of problem solving of which intervention participants appeared capable. Additionally, certain constructs, such as creativity and empathy, stretched across multiple categories of outcomes identified in this study. For instance, creativity was seen both in problem-solving and in proposed helping approaches; empathy was seen in understanding of others’ perspectives and in proposed helping approaches. These outcomes did not arise in other instances, however. Existing ways to measure creativity or empathy may or may not have captured these changes depending on the applications upon which they focused.

It would be difficult, therefore, to find a set of established outcome measures that exactly matched the categories that emerged from this study. This finding suggests that the categories often measured within evaluations of mental health promotion and prevention programs may not always be appropriate within LMICs. Researchers may therefore miss important effects of promotive and preventive mental health programs if existing measures are the only method of measurement. In future evaluations, researchers should consider other means to identify the effects of such programs in LMIC populations. Other strategies could include integrating qualitative methods of identifying outcomes, such as those described in this study, and/or developing or identifying quantitative measures that are specific to the applications of certain skills in a given population.

This concern is particularly relevant for studies that rely on quantitative measures, which generally require researchers to strictly define outcomes *a priori* and often rely on psychometric scales. Most studies that seek to quantify the effects of mental health promotion and prevention programs in LMICs utilize psychometric scales to measure outcomes despite significant difficulties in adaptation and validation across different populations; even the guidelines that exist for doing so have been frequently contradictory (34). Extensive discussion of these challenges is beyond the scope of this manuscript; however, the findings of this study do shed light on the importance of attention to locally relevant outcomes within future studies.

#### Basic skills and desired attitudes may be widespread even without intervention; therefore, higher-level skills and more nuanced attitudes are important to capture

The outcomes identified as emerging to a greater extent in the intervention arm vs. the control arm in this study were frequently high-level skills, while the control group possessed basic levels of a number of skills even without intervention. For instance, the control group girls showed basic perspective-taking skills, while the intervention group girls showed perspective-taking skills that also integrated more nuance, complexity, and higher-order thinking. In order to capture such differences, it is important to identify ways to sensitively capture differences between higher and lower levels of such skills.

Additionally, participants in both the control and intervention arms held similar attitudes about gender: they all believed that gender inequality existed, and that this was unfair. However, boys in the intervention group had a more nuanced, high-level perspective on what the experience for a girl would have been like in the story vs. control boys. This similarity at a basic level but difference at a more nuanced level may reflect that youth in the study, including both control and intervention arms, encountered public gender equality campaigns containing these basic messages that have been recently more prevalent in Bihar (35), as well as throughout India and globally (36). Thus, if a future study wished to capture such differences, measures should take care to go well beyond messages that participants may frequently hear via other sources and delve into higher levels of understanding and perspective-taking.

Thus, particularly if researchers wish to use quantitative psychometric measures, they should anticipate potential for ceiling effects. If a researcher wished to measure the skills and attitudes of youth who attended Youth First via quantitative means, for instance, a measure with a high ceiling that can capture high levels of skills and nuanced attitudes would be ideal. By contrast, if a measure gives its highest possible score to basic skills and basic desired attitudes, it could miss some of these higher-level differences. Researchers should anticipate potential for ceiling effects, particularly as measures are used in new populations, and choose other measures or improve existing measures if they are found to exist. For a robust example of investigating ceiling effects in a psychosocial skills measure within a certain population and adapting accordingly, see (37).

### Recommendations for identifying outcomes in future evaluations of mental health promotion and prevention programs among low- and middle-income countries youth

This study provides useful insights for future efforts that aim to quantify the outcomes of mental health promotion and prevention programs among LMIC youth, particularly for similar school-based interventions. Aligned with the findings discussed above, we propose three recommendations to guide researchers in approaching future evaluations of mental health promotion and prevention programs among LMIC youth.

Researchers should consider:

1. Integrating open-ended, qualitative investigations of outcomes experienced or exhibited by participants, in addition to or instead of quantitative methods. Studies following methods similar to those of the current study, for instance, can assist in identifying what outcomes arise via a mental health promotion and prevention intervention that may not have been anticipated *a priori*. These can then be used to inform the design of evaluations.
2. Identifying target outcomes that are specific to local understandings and applications. Although establishing outcomes based on previous studies from other populations may be easier, doing so may miss the ways in which individuals in different LMIC populations apply skills toward population-specific outcomes.
3. Ensuring that any outcome measures can capture higher-level skills and nuanced attitudes and are sensitive to differences between higher- vs. lower-level skills and nuanced vs. basic desired attitudes. In the case of quantitative measures, this need can in part be mitigated via piloting outcome measures prior to final measure selection, in order to examine distributions within the population prior to intervention.

## Limitations

As with any study, this study has limitations that should be acknowledged. First, as is normal in qualitative data collection, primary researchers were not blinded to study arm during fieldwork or initial stages of thematic analysis. However, the researchers were affiliated with an external research agency that had not been involved during the intervention in order to reduce bias. To mitigate potential bias in the second stages of analysis, the second author examined and performed the same categorization of differences between the intervention and control arms as the first author, blind to the study arm of the participants and blind to the categorizations made by the first author. Initial agreement was high (98%) and categorizations of the two authors were compared and discrepancies resolved with 100% agreement.

A second limitation of the study is that there were differences between the vignettes used for the boys’ vs. girls’ focus group discussions. Although the girls’ and boys’ FGD guides both featured vignettes that described a person of the same gender and age as the participants encountering a problem that was related to social inequality, the specifics of the situation described in the vignette for boys’ vs. girls’ FGDs was different, based on the situations that boys and girls often encounter in the study area. Tailoring the vignette by gender served to help participants to relate to the vignette and find it relevant to their lives. Although this was a necessary step to engage participants with relevant vignettes, differences observed between girls and boys may therefore be attributable to differences in the vignettes rather than actual differences between how girls and boys responded after the intervention. As the study was not meant to focus primarily on differences between girls and boys, this limitation was deemed reasonable to accept, given that it allowed us to ensure that both girls and boys were able to meaningfully engage in discussing a situation that was familiar to them. Any differences that did emerge between boys and girls, however, should therefore be interpreted with caution.

Finally, data collection was undertaken during a time period in which schools were opening and closing due to COVID-related closures. During this time period, some schools in the cRCT were intermittently inaccessible. This situation prompted the inclusion of only schools from the cRCT that were open during the study time period for pragmatic reasons. Although this choice introduces a small possibility of bias into the sample, it was a critical choice to make in order to feasibly conduct the study. We are not aware of any systematic differences among the schools that were open vs. the schools that were closed at this time.

## Conclusion

Overall, adolescents who participated in Youth First exhibited high-level inter- and intra-personal psychosocial skills, understanding, and attitudes, spanning problem-solving, perspective-taking, awareness of strengths, helping behaviors, and more. These findings are particularly promising given Youth First’s potential for scale, and the fact that differences between the intervention and control arm were clearly observable 1.5 years after completion of the intervention. In addition, this study provides insight into the types of outcomes that emerged via the voices of adolescent participants, which can provide direction to future researchers regarding how to establish outcomes of interest when evaluating similar interventions among similar populations. With these insights and the recommendations generated in this paper, we hope that future researchers will be able to better identify and clarify the outcomes of mental health promotion and prevention programs among LMIC youth in the future, leading ultimately to more effective programs that can impact youth mental health on a broad scale.

## Data availability statement

The datasets presented in this article are not readily available. Per our study’s informed consent restrictions agreed with United States and Indian IRBs, primary data consisting of qualitative transcripts cannot be included. The datasets generated for the second phases of the thematic analysis are available by request to the corresponding author, KL.

## Ethics statement

The studies involving human participants were reviewed and approved by the Chesapeake (Advarra) IRB and Sangath IRB. Written informed consent to participate in this study was provided by the participants’ legal guardian/next of kin and participants also provided assent to participate.

## Author contributions

KL collaborated on conceptualization, formal analysis, and funding acquisition, led writing of the original draft of the manuscript and reviewing and editing the manuscript. PC collaborated on the formal analysis and on writing the original draft of the manuscript, and contributed to reviewing and editing the manuscript. LD collaborated on conceptualization and reviewing and editing the manuscript. PP and HS collaborated on project administration and reviewing and editing the manuscript. GA and SL collaborated on the funding acquisition and reviewing and editing the manuscript. All authors contributed to the article and approved the submitted version.
